# Selective deletion of hepatocyte platelet-derived growth factor receptor α and development of liver fibrosis in mice

**DOI:** 10.1186/s12964-018-0306-2

**Published:** 2018-12-03

**Authors:** Beom Jin Lim, Woon-Kyu Lee, Hyun Woong Lee, Kwan Sik Lee, Ja Kyung Kim, Hye Young Chang, Jung Il Lee

**Affiliations:** 1 Department of Pathology, Gangnam Severance Hospital, Yonsei University College of Medicine, Seoul, Republic of Korea; 2 Laboratory of Developmental Genetics, Department of Biomedical Sciences, Inha University College of Medicine, Incheon, Republic of Korea; 3 Department of Internal Medicine, Gangnam Severance Hospital, Yonsei University College of Medicine, Seoul, Republic of Korea; 4 Medical Research Center, Gangnam Severance Hospital, Seoul, South Korea

**Keywords:** Platelet-derived growth factor receptor α, Liver fibrosis, Liver cirrhosis, Hepatocyte, Hepatic stellate cell

## Abstract

**Background:**

*Platelet-derived growth factor receptor α* (*PDGFRα*) expression is increased in activated hepatic stellate cells (HSCs) in cirrhotic liver, while normal hepatocytes express *PDGFRα* at a negligible level. However, cancerous hepatocytes may show upregulation of *PDGFRα*, and hepatocellular carcinoma is preceded by chronic liver injury. The role of PDGFRα in non-cancerous hepatocytes and liver fibrosis is unclear. We hypothesized that upon liver injury, PDGFRα in insulted hepatocytes contributes to liver fibrosis by facilitating intercellular crosstalk between hepatocytes and HSCs.

**Methods:**

Hepatocytes were isolated from normal and thioacetamide (TAA)-induced cirrhotic livers for assessment of *PDGFRα* expression. Conditional knock-out (KO) C57BL/6 mice, in which *PDGFRα* was selectively deleted in hepatocytes, were generated. Liver fibrosis was induced by injecting TAA for 8 weeks. Hep3B cells were transfected with a small interfering RNA (siRNA) (PDGFRα or control) and co-cultured with LX2 cells.

**Results:**

*PDGFRα* expression was increased in hepatocytes from fibrotic livers compared to normal livers. Conditional *PDGFRα* KO mice had attenuated TAA-induced liver fibrosis with decreased HSC activation and proliferation. Immunoblot analyses revealed decreased expression of phospho-p44/42 MAPK in TAA-treated KO mice; these mice also showed almost complete suppression of the upregulation of *mouse double minute 2*. Although KO mice exhibited increased expression of *transforming growth factor (TGF)-β* and *Smad2/3*, this was compensated for by increased expression of inhibitory *Smad7*. LX2 cells co-cultured with PDGFRα siRNA-infected Hep3B cells showed decreased *PDGFRα*, *α smooth muscle actin*, *collagen α1(I)*, *TGFβ*, and *Smad2/3* expression. LX2/*PDGFRα*-deleted hepatocyte co-culture medium showed decreased PDGF-BB and PDGF-CC levels.

**Conclusions:**

Deletion of *PDGFRα* in hepatocytes attenuated the upregulation of *PDGFRα* in HSCs after TAA treatment, resulting in decreased liver fibrosis and HSC activation. This suggests that in the event of chronic liver injury, PDGFRα in hepatocytes plays an important role in liver fibrosis by affecting *PDGFRα* expression in HSCs.

## Background

Liver fibrosis is a multicellular response to liver injury in which hepatic stellate cells (HSCs) are responsible for 90% of extracellular matrix (ECM) production [1–5]. Liver fibrosis upon acute liver injury may be beneficial by providing mechanical stability and facilitating the restoration of hepatic architecture and function. However, repetitive chronic liver insults result in liver fibrosis, which may progress to cirrhosis [3, 6, 7], the major contributing factor to development of hepatocellular carcinoma (HCC) [8].

Upon liver injury, HSCs are activated by cytokines, including platelet-derived growth factors (PDGFs), which are potent mitogens [9, 10]. PDGFs exist as five isoforms: PDGF-AA, PDGF-AB, PDGF-BB, PDGF-CC, and PDGF-DD. The cellular effects of PDGFs require the function of cell surface receptor tyrosine kinases comprising platelet-derived growth factor receptor (PDGFR)α and PDGFRβ subunits [11, 12]. Elevated *PDGFR* expression is observed in human cardiac fibrosis after myocardial infarct, pulmonary fibrosis, kidney fibrosis, and liver fibrosis [13–16].

*PDGFR* expression is very low in the normal adult liver but is significantly upregulated in HSCs in cirrhotic liver [17]. PDGFRα participates in liver fibrosis, as evidenced by increased PDGFRα expression in cirrhotic human livers, human cell lines, and a mouse model of liver fibrosis [18, 19]. Stimulation of *PDGFRα* expression in HSCs induces their activation and proliferation [16, 20–23], and blocking of *PDGFRα* expression suppresses HSC proliferation [24]. In addition, stimulation of *PDGFRα* expression by excessive production of PDGF-CC induces liver cirrhosis and HCC in PDGF-C transgenic (Tg) mice [25, 26]. *PDGFRα* expression in the liver is markedly increased in PDGF-C Tg mice, although the responsible cell type is unclear.

Normal adult hepatocytes show negligible *PDGFRα* expression. However, hepatocytes in embryos may have enhanced *PDGFRα* expression, which later is markedly attenuated [27]. Therefore, the role of *PDGFRα* expression in adult hepatocytes in liver fibrosis is unknown. However, the increased *PDGFRα* expression in HCC subsets in the absence of background liver cirrhosis suggests that abnormal hepatocytes overexpress *PDGFRα* [28].

Our preliminary study demonstrated that *PDGFRα* expression was increased in not only stimulated HSCs but also injured hepatocytes. We hypothesized that PDGFRα in hepatocytes in the setting of chronic liver injury plays an important role in liver fibrosis by facilitating intercellular crosstalk between hepatocytes and HSCs.

We report for the first time that conditional abolition of *PDGFRα* expression in hepatocytes attenuates chemically induced liver fibrosis by suppressing the upregulation of *PDGFRα* and TGFβ in HSCs, reducing their activation and proliferation.

## Methods

### Generation of *PDGFRα* conditional KO mice

Homozygous PDGFRα^loxp/Loxp^ mice with targeted deletion of exons 1 to 4 and albumin (alb)-Cre mice, both on the C57BL/6 background, were obtained from Jackson Laboratories (Bar Harbor, ME). PDGFRα^loxp/Loxp^ mice were crossbred with Alb-Cre mice to obtain offspring carrying PDGFRα^loxp/wt^;Alb-Cre. Next, PDGFRα^loxp/wt^;Alb-Cre were bred to PDGFRα^loxp/Loxp^ mice, resulting in PDGFRα^loxp/Loxp^;Alb-Cre^+/−^ or KO mice. Mice of all other genotypes, including PDGFRα^loxp/Loxp^;Alb-Cre^−/−^ and PDGFRα^loxp/wt^;Alb-Cre^−/−^, are referred to as wild-type (WT) controls.

### Animals and thioactetamide-induced liver fibrosis

All experiments on mice were approved by the Institutional Animal Use and Care Committee (IACUC) at Gangnam Severance Hospital, Yonsei University College of Medicine (permit number 0031) in accordance with the recommendations and restrictions of the IACUC, which follows the guidelines of the United States National Institutes of Health.

Male 8-week old C57BL/6 (WT) and PDGFRα conditional KO mice (*PDGFRα* KO) were used. The animals were housed with a 12 h light:dark cycle and fed standard chow. For the fibrosis groups, thioacetamide (TAA) (300 mg/kg body weight) was injected intraperitoneally into male WT and PDGFRα KO mice during the light cycle twice weekly for 8 weeks. One week after the final TAA treatment, the mice were fasted for 14–16 h with free access to water and subsequently euthanized. Untreated age-matched WT or *PDGFRα* KO male mice were euthanized after the fast.

The animal experiment groups were as follows: WT-control (*n* = 5), untreated WT mice; KO-control (*n* = 5), untreated *PDGFRα* conditional KO mice; WT-TAA (*n* = 5), WT mice treated with TAA for 8 weeks; and KO-TAA (*n* = 5), *PDGFRα* conditional KO mice treated with TAA for 8 weeks.

### Hepatocyte and HSC isolation

Hepatocytes and HSCs were isolated from WT and WT-TAA mice by an in situ collagenase perfusion method as described previously with modifications [29]. Protein and RNA were extracted from the isolated hepatocytes for analyses.

### HCC tissue

Freshly frozen liver-tissue specimens from livers resected due to HCC or metastatic liver cancer were used. The liver specimens were archived at the Tissue Bank of Yonsei University College of Medicine, Gangnam Severance Hospital with the patients’ consent. The non-tumor sites of liver specimens with hepatitis but not liver cirrhosis as determined via pathologic evaluation (*n* = 7) were compared to liver specimens from metastatic liver cancer patients (n = 7) that appeared normal on pathologic assessment. This study was approved by the Institutional Review Board of Gangnam Severance Hospital, Yonsei University College of Medicine, Seoul, Republic of Korea (3–2015–0045).

### Immunohistochemical analyses and terminal dUTP nick end-labeling assay

Liver-tissue specimens were stained with Masson’s trichrome and the fibrosis ratio was calculated using an image analysis system as described previously with modifications [30]. The total area was calculated as the sum of the area of the microscopic fields, including parenchyma and fibrosis. For each slide, the area of fibrosis was evaluated in 20 consecutive fields at a magnification of × 200, and averaged.

Sections of mouse liver tissue specimens were immunostained using a mouse anti-human alpha-smooth muscle actin (αSMA) primary antibody (DAKO, Carpinteria, CA). Detection of the primary antibody was carried out by the immunoperoxidase technique using an ABC Kit (Vector Laboratories) as a measure of HSC activation. Peroxidase activity was determined by reaction with diaminobenzidine tetrahydrochloride (DAB). Data are total numbers of αSMA-positive cells present in 10 high-power fields (× 400) per sample.

Apoptosis was evaluated by terminal dUTP nick end-labeling (TUNEL) assay. The TUNEL assay detects fragmentation of nuclear DNA during apoptotic cell death in situ, and was performed using an ApopTag Peroxidate In Situ Apoptosis Detection Kit (Millipore, USA). For each slide, the area of immunostained apoptotic nuclei by 3’-OH-end labeling of fragmented DNA was evaluated in 20 consecutive fields at a magnification of × 200, and averaged.

Sections of human liver-tissue specimens were immunostained with a mouse anti-PDGFRα antibody (Santa Cruz Biotechnology, Santa Cruz, CA) and assessed at a magnification of × 400.

### siRNA transfection

Transient knockdown assays were performed using DharrmaFECT 1 (Dharmacon, Lafayette, CO), according to the manufacturer’s instructions. PDGFRα (#5156) and control (D-001206-13) siRNAs were purchased from Dharmacon.

### Cell lines and in vitro experiments

Hep3B (KCLB #88064) and LX2 (cat #SCC064) cells were purchased from the Korean Cell Line Bank (Seoul, Republic of Korea), and EMD Millipore (Temecula, CA), respectively, and maintained as described previously [31, 32].

Cells were cultured using hanging cell-culture inserts (1 μm pore size, Falcon) to separate cell populations. Wells and inserts with medium were allowed to stabilize for 24 h at 37 °C according to the manufacturer’s recommendations. Hep3B cells were seeded in the insert (3 × 10^3^ cells/cm^2^) and allowed to attach overnight in Dulbecco’s modified Eagle’s medium (DMEM) with 10% fetal bovine serum (FBS). The following day, siRNA (PDGFRα or control) was transfected into Hep3B cells using DharmaFECT 1 (Dharmacon, Lafayette, CO), according to the manufacturer’s instructions. Briefly, LX2 cells (3 × 10^5^/cm^2^) were seeded on a six-well plate and incubated for 24 h. Hep3B cells were plated separately on a culture insert and transfected with the PDGFRα or control siRNA. After a 6 h stabilizing period, the insert containing treated Hep3B cells was placed above the plate with LX cells and incubated for a further 24 h. LX2 Cells and supernatants were harvested and recovered for further analyses.

### Cell viability assay

The 3-(4,5-dimethylthiazol-2-yl)-2,5-diphenyltetrazolium bromide (MTT) assay was used to evaluate the proliferation of LX cells. Following co-culture for 24 h, 5 mg/mL MTT was added to the culture medium (Sigma-Aldrich, St. Louis, MO) and incubated for 4 h at 37 °C. The supernatant was removed, the cells were treated with 150 μL/well dimethyl sulfoxide, and the absorption at 470 nm was measured. The assay was carried out in triplicate and each experiment was repeated at least three times. Data are percentages of surviving cells relative to the control.

### RNA isolation and real-time polymerase chain reaction

Total RNA was extracted from frozen whole livers or isolated cells using TRIzol reagent (Invitrogen, Carlsbad, CA) or Qiagen mini columns (Qiagen Inc. Valencia, CA) according to the manufacturer’s protocol. RNA concentrations were quantified by spectrophotometry. RNA integrity was assessed by agarose gel electrophoresis and ethidium bromide staining. The RNA samples were diluted in RNase-free water and stored at − 70 °C until use. Five micrograms of RNA were reverse-transcribed using the RNA PCR Kit, version 1.2 (TaKaRa Bio Inc., Japan) according to the manufacturer’s recommendations. Oligonucleotide primers and a TaqMan probe for *PDGFRα*; *TGF-β*; *Smad 2*, *3*, *and 7; collagen α1(I)* (*Col1α(I)*); *αSMA; bax; bcl-2;* and *MDM2* were used, with *18S* as the internal control. The probes were obtained from Applied Biosystems (Perkin-Elmer/PE Applied Biosystems, Foster City, CA). The TaqMan probe was labeled at the 5′-end with the reporter dye FAM and at the 3′-end with a minor groove binder (MGB) nonfluorescent quencher. Quantitative polymerase chain reaction (qPCR) was performed in triplicate for each sample on a Step One Plus Real Time System (Applied Biosystems). Each 20 μL reaction contained 10 μL TaqMan Fast Universal Master Mix (Applied Biosystems, Darmstadt, Germany), 1 μL Gene Expression Mix, and 2 μL cDNA diluted in 7 μL RNase-free water. The thermal cycler conditions were 20 s at 95 °C, followed by 40 cycles of 5 s at 95 °C and 20 s at 60 °C. Fold changes in the expression of target genes relative to the endogenous 18S control were calculated as described previously [33].

### Protein extraction and immunoblotting

Whole-liver lysates were prepared in Triton-X 100 lysis buffer containing protease inhibitors and protein concentrations were quantified using the Bradford method with bovine serum albumin (BSA) as the standard. Resolution by SDS-PAGE was followed by immunoblotting using the following antibodies: rabbit anti-PDGFRα (#3164), rabbit anti-phospho-p44/42 MAPK (#9101), rabbit anti-p44/42 MAPK (#9102), rabbit anti-β actin (#4(a967), all from Cell Signaling (Danvers, MA) and anti-phospho-Smad3 (#ab52903) from Abcam (Cambridge, MA). Epitope-primary antibody complexes were detected using species-specific secondary antibodies conjugated to horseradish peroxidase (HRP) followed by enhanced chemiluminescence (ECL) (Thermo Fisher Scientific Pierce, IL).

### Determination of PDGF-CC and -BB levels in culture medium

The levels of PDGF-CC and PDGF-BB in culture medium were quantified using a Quantikine enzyme-linked immunosorbent assay (ELISA) Kit (R&D Systems, Minneapolis, MN) according to the manufacturer’s instructions.

### Statistical analyses

Results are shown as means ± standard errors of the mean (SEMs). Data were subjected to nonparametric analyses (Kruskal–Wallis or Mann–Whitney test) or one-way analysis of variance (ANOVA) with Tukey’s post hoc test. A value of *P* < 0.05 was considered indicative of statistical significance. All calculations were performed in IBM SPSS Statistics version 23 for Windows.

## Results

### *PDGFRα* deletion in hepatocytes attenuated the TAA-induced increase in *PDGFRα* expression in the whole liver

The level of *PDGFRα* expression is increased in fibrotic liver due to its overexpression in HSCs [19, 34]. Non-insulted hepatocytes scarcely express *PDGFRα* [19].

**Fig. 1:**
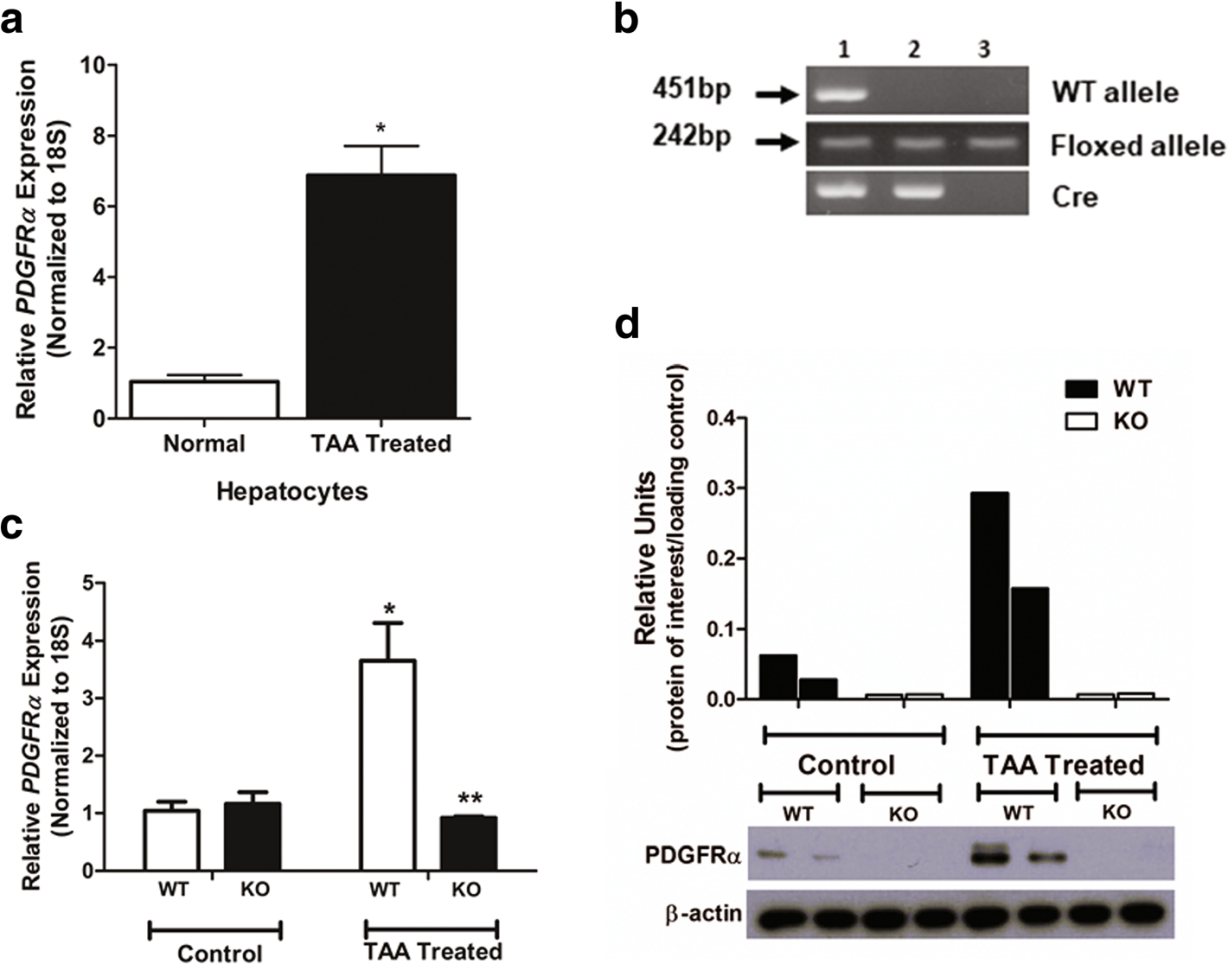
Attenuation of *PDGFRα* expression in TAA-treated livers after conditional deletion of *PDGFRα* in hepatocytes. **a** *PDGFRα* in hepatocytes. **b** Identification of hepatocyte *PDGFRα*-deleted knockout (KO) mice by genotyping PCR (lane 2). **c** *PDGFRα* expression in the liver of wild-type (WT) and hepatocyte *PDGFRα*-deleted KO mice (**d**) Western blotting analyses; densitometry values normalized to that of β-actin are shown. *P < 0.05, compared to that of TAA-untreated WT mice. **P < 0.05, compared to WT mice under identical conditions

Hepatocytes from TAA-treated livers showed increased *PDGFRα* expression compared to those from normal livers (Fig. 1a). To address the role of hepatocyte PDGFRα in liver fibrosis, we generated hepatocyte-specific conditional *PDGFRα* KO mice (Fig. 1b). The non-insulted livers (control) of WT and conditional KO mice showed no significant differences in *PDGFRα* expression (Fig. 1c). However, the livers of TAA-treated WT mice, but not those of TAA-treated KO mice, had enhanced *PDGFRα* expression (Fig. 1c).

Immunoblot analyses of the whole liver showed an increased PDGFRα protein level in TAA-treated WT mice, and a significantly reduced PDGFRα protein level in TAA-treated KO mice (Fig. 1d).

### *PDGFRα* deletion in hepatocytes alleviated TAA-induced HSC activation and liver fibrosis

**Fig. 2:**
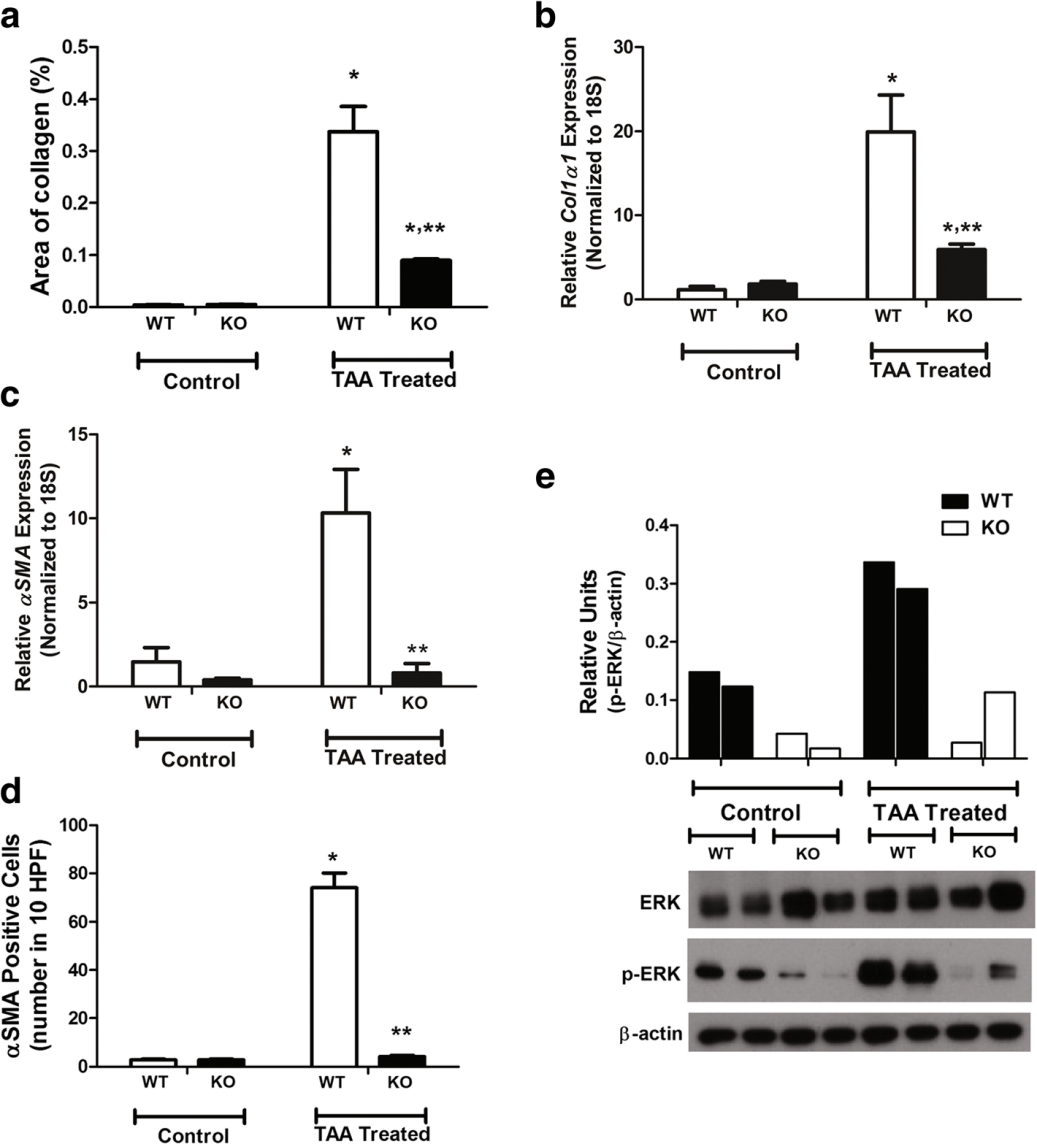
Suppression of TAA-induced liver fibrosis by hepatocyte platelet-derived growth factor receptor (PDGFR) α deletion. **a** Area of fibrosis. **b** Expression of (**b**) C*ol1α(I)* and **c** *αSMA*. **d** Number of αSMA-positive cells. **e** Western blotting analyses; densitometry values are shown. *P < 0.05, compared to TAA-untreated WT mice. **P < 0.05, compared to WT mice under identical conditions

The fibrotic area in the liver was increased by TAA in both WT and KO mice (Fig. 2a). However, *PDGFRα* conditional KO mice showed significantly attenuated liver fibrosis compared to WT mice (Fig. 2a). Quantitative analyses of *col1α(I)* expression yielded a result comparable with the histologic findings (Fig. 2b).

The TAA-induced increase in the expression of *αSMA*, a marker of HSC activation, was significantly abolished by deletion of *PDGFRα* (Fig. 2c). The number of αSMA-positive cells was significantly increased and decreased by TAA in the liver of WT and KO mice, respectively (Fig. 2d).

### Deletion of *PDGFRα* in hepatocytes attenuates ERK activation

ERK is a downstream signaling factor involved in PDGF-induced activation of PDGFR [35]. Immunoblot analyses of phospho-p44/42 MAPK (ERK1/2) revealed increased phosphorylation of ERK1/2 in the livers of WT and KO TAA-treated mice compared to the controls. However, KO mice showed significantly attenuated phosphorylation of ERK1/2 compared to WT TAA-treated mice (Fig. 2e).

### Deletion of *PDGFRα* did not influence hepatocyte apoptosis

**Fig. 3:**
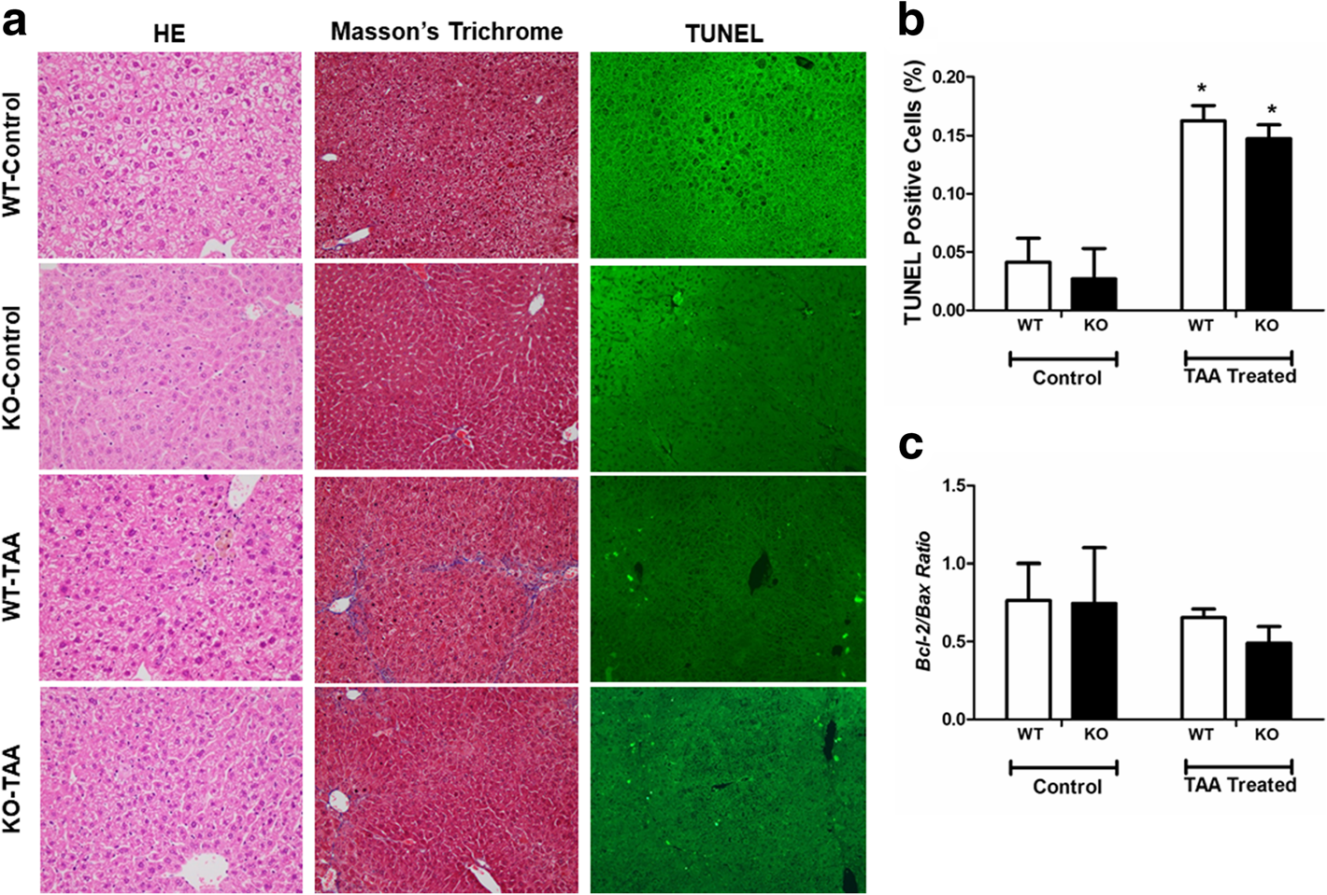
Effects of conditional *PDGFRα* deletion on hepatocyte morphology and apoptosis. **a** Hematoxylin and eosin (HE) staining (× 400), Masson’s trichrome staining (× 200), and TUNEL staining (× 400) of the liver. **b** Area of immunostained liver. **c** Liver *Bcl-2: Bax* expression ratios. *P < 0.05, compared to TAA-untreated WT mice. **P < 0.05, compared to WT mice under identical conditions

TAA treatment increased the number of cells displaying nuclear fragmentation compared to the control. Deletion of *PDGFRα* in hepatocytes did not affect apoptosis irrespective of TAA treatment, similar to the WT control (Fig. 3a, b). Bcl-2 inhibits apoptosis whereas Bax promotes it [36], and the Bcl-2/Bax ratio is a marker of survival after apoptotic stimuli [37]. The liver Bcl-2/Bax ratio did not differ significantly between WT and KO mice after TAA treatment (Fig. 3c).

### Effect of hepatocyte *PDGFRα* deletion on TGFβ signaling

**Fig. 4:**
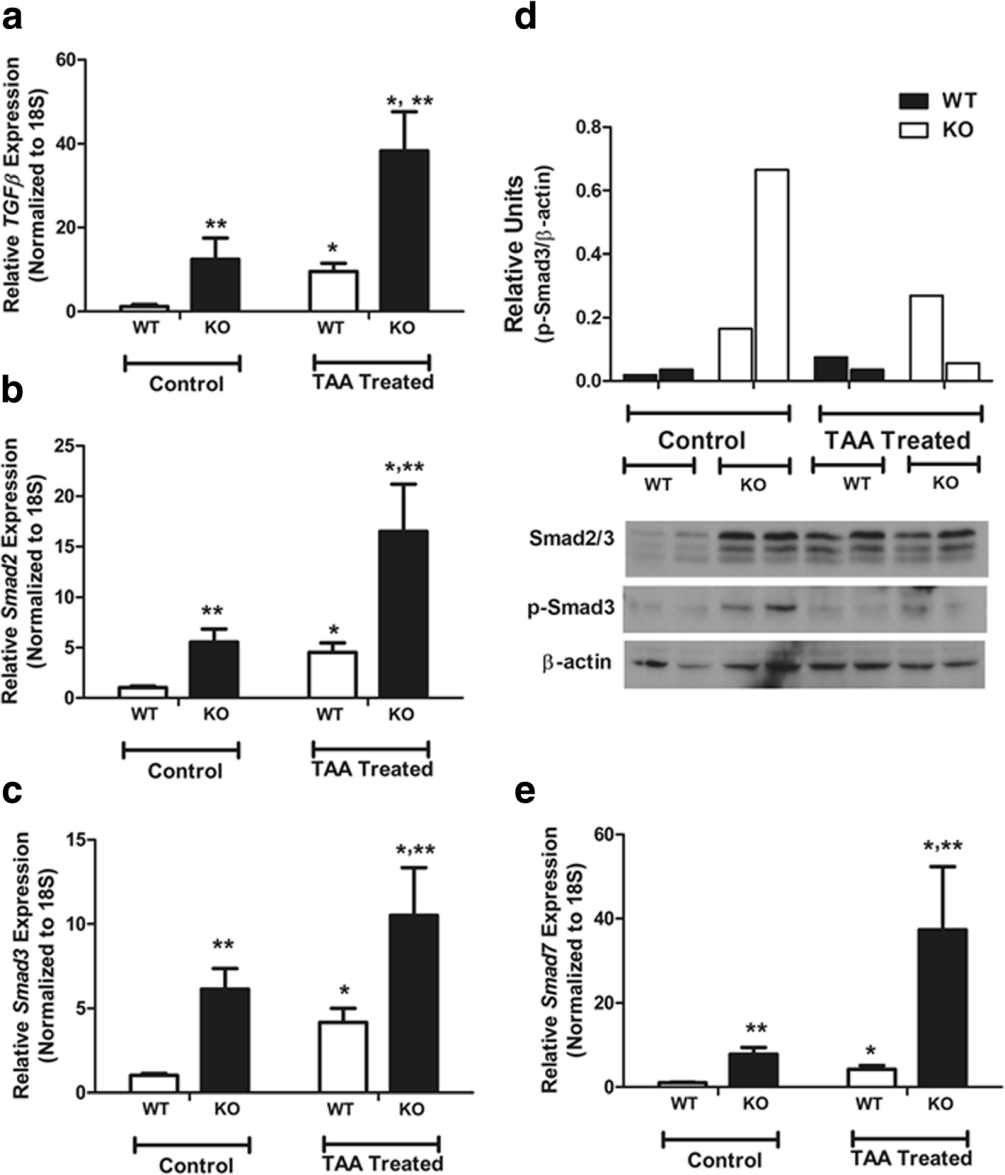
Effect of hepatocyte *PDGFRα* deletion on TGF-β signaling in TAA-treated mouse liver. Expression of (**a**) *TGFβ* (**b**), *Smad2* (**c**), and *Smad3*. **d** Western blotting analyses of Smads; densitometry values normalized to that of β-actin are shown. **e** Expression of inhibitory *Smad7*. *P < 0.05, compared to TAA-untreated WT mice. **P < 0.05, compared to WT mice under identical conditions

Activation of PDGFR by excessive PDGF ligands results in upregulation of the TGFβ/Smad signaling pathway to facilitate HSC proliferation and collagen production [38]. In this study, deletion of *PDGFRα* in hepatocytes resulted in increased *TGFβ* expression in normal and TAA-treated livers compared to the WT (Fig. 4a). Downstream signaling of TGFβ and *Smad2*/*3* was also upregulated by deletion of *PDGFRα* in hepatocytes in both normal and TAA-induced fibrotic livers compared to the WT (Fig. 4b, c). The protein level of Smad3 showed a similar trend to the mRNA level (Fig. 4d). *Smad7* was significantly upregulated in the liver of conditional *PDGFRα*-deleted mice; this likely compensated for the effects of enhanced TGF-β signaling (Fig. 4d).

### Deletion of *PDGFRα* in hepatocytes blocked upregulation of MDM2

**Fig. 5:**
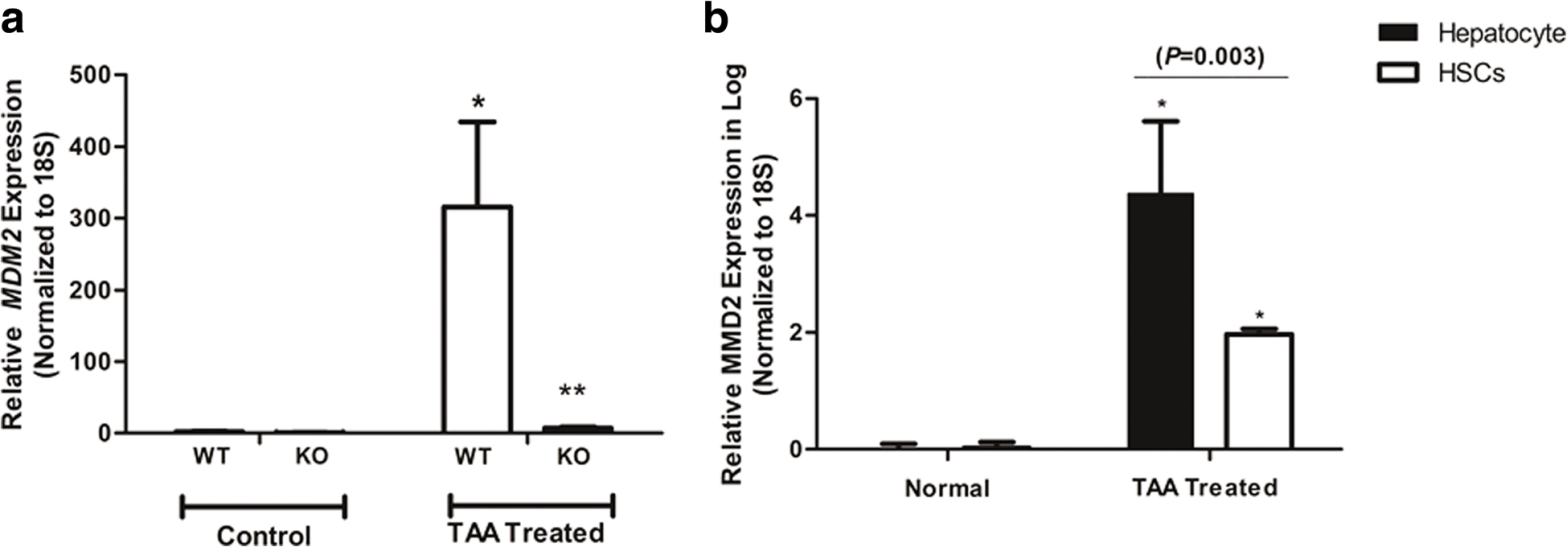
Effect of hepatocyte *PDGFRα* deletion on *MDM2* upregulation after TAA-induced liver injury. **a** *MDM2* expression in the liver normalized to that of the 18S rRNA gene. *P < 0.05, compared to TAA-untreated WT mice. **P < 0.05, compared to WT mice under identical conditions. **b** Expression of *MDM2* in hepatocytes and HSCs. *P < 0.05, compared to normal liver cells

MDM2 is a negative regulator of p53 and is considered an oncogene [39, 40]. Activation of PDGRα independently of PDGF results in activation of MDM2 and suppression of p53 [41, 42]. TAA-induced liver injury was accompanied by upregulation of *MDM2* in the liver; this was blocked by deletion of *PDGFRα* in hepatocytes (Fig. 5a).

To speculate on the cellular components responsible for the TAA-induced increase in *MDM2* expression, hepatocytes and HSCs were isolated from TAA-treated livers and *MDM2* expression was evaluated. Although both hepatocytes and HSCs from injured livers showed increased *MDM2* expression, the magnitude of the upregulation was significantly greater in hepatocytes than in HSCs (Fig. 5b).

### Co-culture with *PDGFRα*-deleted Hep3B cells decreased *PDGFRα* expression in, and suppressed the activation of, LX2 cells

**Fig. 6:**
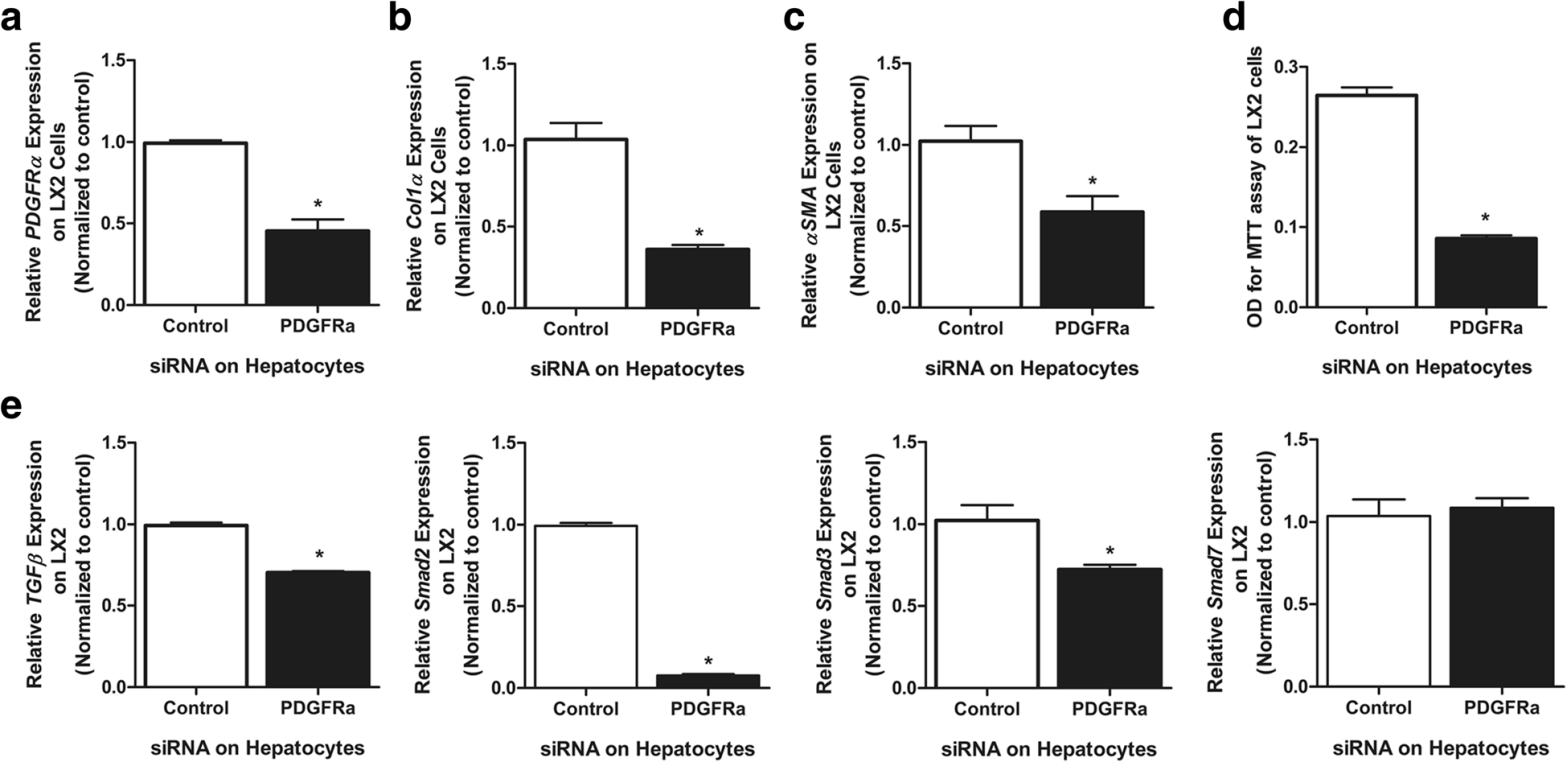
Changes in LX2 cells co-cultured with Hep3B cells with or without *PDGFRα* deletion. **a** Expression of (**a**) *PDGFRα*, **b** *col1α(I)* and **c** αSMA in LX2 cells co-cultured with Hep3B cells with or without *PDGFRα* silencing. **d** MTT assay results. **e** Expression of *TGFβ*, *Smad2*, *Smad3*, and *Smad7* in LX2 cells. *P < 0.05, compared to LX2 cells co-cultured with PDGFRα-preserved Hep3B cells

Hep3B cells have elevated *PDGFRα* expression [19, 43]. Culture of LX2 cells with PDGFRα-silenced Hep3B cells attenuated *PDGFRα* expression in the former (Fig. 6a). They had decreased *αSMA* and *col1α(I)* expression compared to that on LX2 cells, co-cultured with Hep3B cells with normally expression PDGFRα (Fig. 6b, c).

LX2 cells were co-cultured with *PDGFRα*-silenced or control Hep3B cells for 24 h. The proliferation of LX2 cells was reduced by co-culture with *PDGFRα*-silenced Hep3B cells (Fig. 6d).

### TGF β signaling in LX2 cells co-cultured with *PDGFRα*-silenced Hep3B cells

LX2 cells cultured with *PDGFRα*-silenced Hep3B cells exhibited attenuated expression of *TGFβ*, and S*mad2/3* (Fig. 6e). However, expression of *Smad7* was not downregulated by co-culture with *PDGFRα*-silenced Hep3B cells (Fig. 6e).

### PDGF ligands in *PDGFRα*-silenced Hep3B cell co-culture medium

**Fig. 7:**
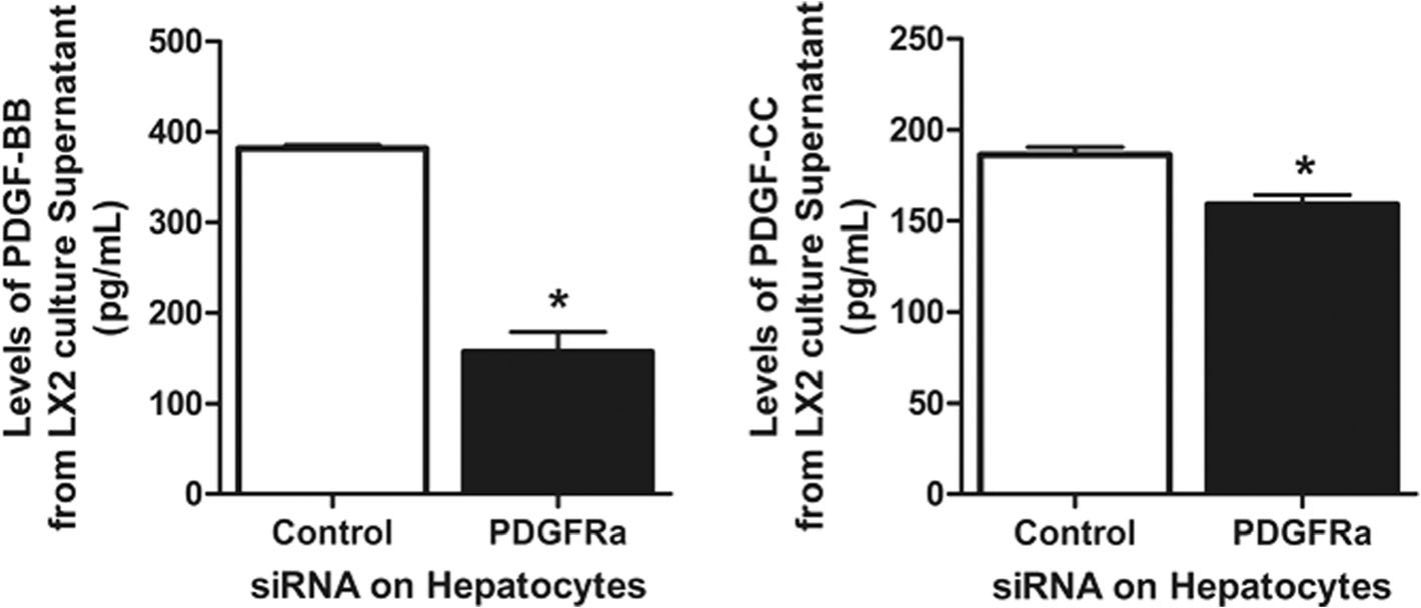
Levels of PDGF-BB and -CC in Hep3B and LX2 co-culture medium. *P < 0.05, compared to LX2 cells co-cultured with *PDGFRα*-preserved Hep3B cells

The levels of PDGF-BB and PDGF-CC were significantly reduced in the co-culture medium of LX2 cells with *PDGFRα*-silenced Hep3B cells, and the reduction in the PDGF-BB level was of greater magnitude than that in the PDGF-CC level (Fig. 7).

### *PDGFRα* expression in human liver with hepatitis without advanced fibrosis

**Fig. 8:**
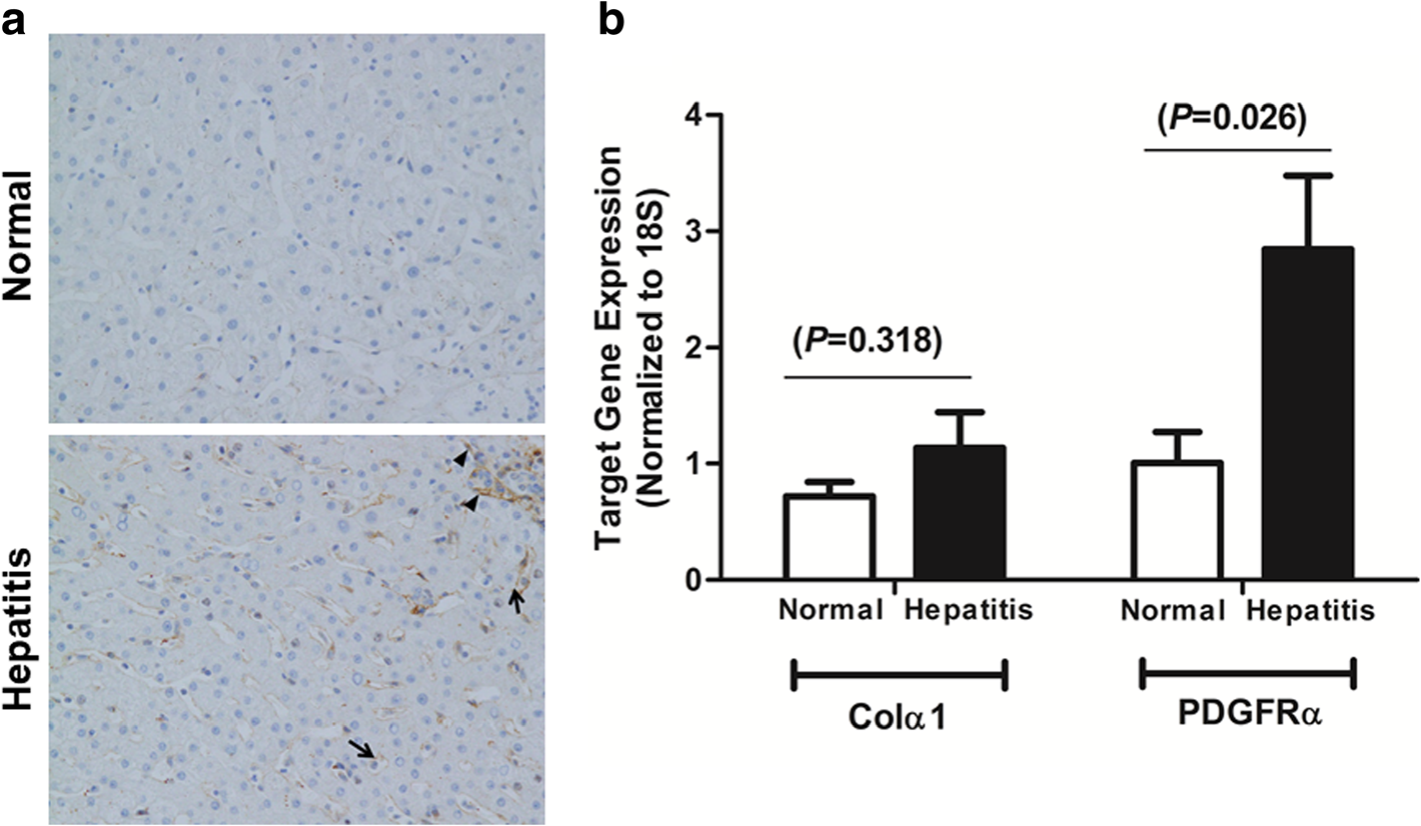
Assessment of PDGFRα expression in the human liver with hepatitis without liver fibrosis. **a** Immunohistochemistry of liver specimens with hepatitis showed the presence of PDGFRα at the hepatocyte membrane (arrows) and in nonparenchymal cells (arrowheads), whereas normal-liver specimens showed no PDGFRα staining. Original magnification, × 400. **b** Whole-cell lysates from pathologically proven hepatitis tissue without significant fibrosis (*n* = 7) were examined for *col1α(I)* and *PDGFRα* mRNA levels compared to those in normal liver tissue (*n* = 7)

Expression of *PDGFRα* was examined in normal human liver and human liver with hepatitis but not advanced fibrosis. Immunohistochemistry showed expression of PDGFRα at the hepatocyte membrane and in nonparenchymal cells in the liver with hepatitis, whereas the normal liver exhibited no PDGFRα staining (Fig. 8a).

Whole-cell lysates from livers with pathologically proven hepatitis but not advanced fibrosis (*n* = 7) were examined for *col1α(I)* and *PDGFRα* expression and compared to that in normal liver tissue (*n* = 7). Livers with hepatitis but not significant liver fibrosis showed elevated PDGFRα expression compared to the normal livers, but *col1α(I)* expression was not significantly different (Fig. 8b).

## Discussion

*PDGFRα* expression is markedly elevated in chronic liver injury and enhanced PDGFRα activation contributes to liver fibrosis [19, 34]. The proliferation of *PDGFRα*-positive HSCs during liver fibrosis can be suppressed by blocking of *PDGFRα* in HSCs [24]. While only PDGFRα in HSCs has been thought to play a role in liver fibrosis, we report that PDGFRα is upregulated in injured hepatocytes, which contributes to HSC proliferation, resulting in liver fibrosis. Deletion of *PDGFRα* in hepatocytes significantly attenuated TAA-induced liver fibrosis. PDGFRα in hepatocytes plays an important role in liver fibrosis by inducing HSC activation and proliferation; in vitro, HSCs co-cultured with *PDGFRα*-deleted hepatocytes exhibited attenuated activation and decreased collagen production.

Normal adult hepatocytes express a low level of *PDGFRα*, the role of which in hepatocytes in liver fibrosis was unclear. However, *PDGFRα* is reportedly expressed in cancerous hepatocytes, and facilitates the proliferation and migration of HCC cells, which are related to invasion and metastasis [44, 45]. Hepatocytes expressing dominant-negative *PDGFRα* revealed decreased TGF-β-induced migration and tumor formation [44]. Therefore, our data suggest that PDGFRα in injured hepatocytes also contributes to liver fibrosis, the most important risk factor for HCC.

siRNA KO of *PDGFRα* in hepatocytes resulted in decreased PDGF-BB and PDGF-CC levels in co-culture medium compared to co-culture of non-KO hepatocytes. Because activated HSCs are possible sources of PDGF ligands [46], and HSC activation was attenuated by *PDGFRα* deletion in hepatocytes, the reduced PDGF-BB and PDGF-CC levels were likely due to decreased production by HSCs. In addition, the decrement in the PDGF-BB level was of greater magnitude than that in the PDGF-CC level. PDGF-BB is reportedly the most potent mitogen for HSC activation, and some animal models of fibrosis, such as the bile-duct ligation model, show upregulation of PDGF-BB and PDGF-CC, but the latter to a lesser extent [47]. The mechanism by which PDGFRs on hepatocytes are activated is unclear. Increased ERK activation was detected in the livers of TAA-treated WT and KO mice, suggesting that PDGF ligands mediate PDGFR activation upon liver insult; the *PDGFRα* KO mice exhibited attenuated ERK activation. However, upregulation of *MDM2*, which is activated at the time of PDGF-independent PDGFR activation, was almost completely blocked by *PDGFRα* deletion in hepatocytes when WT mice liver showed upregulation of *MDM2* after TAA treatment. Although both hepatocytes and HSCs from TAA-treated livers had elevated *MDM2* expression, the upregulation in hepatocytes was about one thousand-fold higher than that in HSCs. Therefore, PDGFRα in hepatocytes might be predominantly activated by non-PDGF mediated mechanisms; this warrants further research.

Elevated levels of TGFβ are seen in liver fibrosis and TGFβ overexpression results in liver fibrosis [48, 49]. TGFβ binds to cell surface receptors to initiate intracellular signal transduction pathways, including activation of Smad proteins [50–52]. Smad2 and Smad3 form complexes with Smad4 to activate gene expression. Smad7, an inhibitory Smad, disrupts receptor activation of Smad2/3, which inhibits TGFβ signaling in a negative feedback loop [53–55]. Activation of PDGFRα ligands by excess PDGF-C upregulates TGFβ/Smad3 signaling to facilitate liver fibrosis and blocking of Smad3 signaling attenuates PDGF-C–induced liver fibrosis [38]. Deletion of *PDGFRα* in hepatocytes results in upregulation of *TGFβ* and *Smad2/3* expression in the whole liver. However, this increase in *TGFβ* and *Smad2/3* expression is offset by overexpression of *Smad7*, resulting in attenuation of TAA-induced liver fibrosis. The cellular source of elevated *TGFβ*, *Smad2/3*, and *Smad7* in the liver after hepatocyte *PDGFRα* deletion is unclear. However, co-culture of *PDGFRα*-deleted hepatocytes and normal HSCs resulted in significant upregulation of *TGFβ* and *Smad2/3* expression in HSCs and maintenance of *Smad7* expression.

Particles of injured hepatocytes, as apoptotic bodies, promote secretion of proinflammatory and fibrogenic cytokines from neighboring inflammatory cells such as macrophages [56]. In addition, damage-associated molecular patterns (DAMPs) have been suggested to be released from injured hepatocytes; this may induce liver fibrosis [57]. It can be speculated that PDGFRα in injured hepatocytes induces liver fibrosis by promoting the release of DAMPs and activating HSCs. Although *PDGRα*-deleted HCC cells exhibit enhanced apoptosis [43], this was not so for non-cancerous hepatocytes in this study.

## Conclusions

Our findings suggest that although *PDGFRα* expression in normal hepatocytes is negligible, it is upregulated by liver insult and plays a vital role in liver fibrosis by facilitating the activation and proliferation of HSCs.

## Abbreviations

Alb: Albumin
Col1α(I): Collagen 1α(I)
DAMP: Damage-associated molecular pattern
ECM: Extracellular matrix
HCC: Hepatocellular carcinoma
HSC: Hepatic stellate cell
KO: Knockout
MDM2: Mouse double minute 2
MTT: Methyl thiazolyl tetrazolium
PDGF: Platelet-derived growth factor
PDGFR: Platelet-derived growth factor receptor
TAA: Thioacetamide
Tg: Transgenic
TGFβ: Transforming growth factor beta
TUNEL: Terminal dUTP nick end-labeling
WT: Wild-type
αSMA: Alpha-smooth muscle actin
